# CG hypermethylation of the *bHLH39* promoter regulates its expression and Fe deficiency responses in tomato roots

**DOI:** 10.1093/hr/uhad104

**Published:** 2023-05-12

**Authors:** Huihui Zhu, Guanghao Han, Jiayi Wang, Jiming Xu, Yiguo Hong, Li Huang, Shaojian Zheng, Jianli Yang, Weiwei Chen

**Affiliations:** State Key Laboratory of Plant Physiology and Biochemistry, College of Life Sciences, Zhejiang University, Hangzhou 310058, China; Research Centre for Plant RNA Signaling, College of Life and Environmental Sciences, Hangzhou Normal University, Hangzhou 311121, China; State Key Laboratory of Plant Physiology and Biochemistry, College of Life Sciences, Zhejiang University, Hangzhou 310058, China; State Key Laboratory of Plant Physiology and Biochemistry, College of Life Sciences, Zhejiang University, Hangzhou 310058, China; Research Centre for Plant RNA Signaling, College of Life and Environmental Sciences, Hangzhou Normal University, Hangzhou 311121, China; Laboratory of Cell & Molecular Biology, Institute of Vegetable Science, Zhejiang University, Hangzhou 310058, China; State Key Laboratory of Plant Physiology and Biochemistry, College of Life Sciences, Zhejiang University, Hangzhou 310058, China; State Key Laboratory of Plant Physiology and Biochemistry, College of Life Sciences, Zhejiang University, Hangzhou 310058, China; State Key Laboratory of Plant Physiology and Biochemistry, College of Life Sciences, Zhejiang University, Hangzhou 310058, China; Research Centre for Plant RNA Signaling, College of Life and Environmental Sciences, Hangzhou Normal University, Hangzhou 311121, China

## Abstract

Iron (Fe) is an essential micronutrient for all organisms, including plants, whose limited bioavailability restricts plant growth, yield, and nutritional quality. While the transcriptional regulation of plant responses to Fe deficiency have been extensively studied, the contribution of epigenetic modulations, such as DNA methylation, remains poorly understood. Here, we report that treatment with a DNA methylase inhibitor repressed Fe deficiency-induced responses in tomato (*Solanum lycopersicum*) roots, suggesting the importance of DNA methylation in regulating Fe deficiency responses. Dynamic changes in the DNA methylome in tomato roots responding to short-term (12 hours) and long-term (72 hours) Fe deficiency identified many differentially methylated regions (DMRs) and DMR-associated genes. Most DMRs occurred at CHH sites under short-term Fe deficiency, whereas they were predominant at CG sites following long-term Fe deficiency. Furthermore, no correlation was detected between the changes in DNA methylation levels and the changes in transcript levels of the affected genes under either short-term or long-term treatments. Notably, one exception was CG hypermethylation at the *bHLH39* promoter, which was positively correlated with its transcriptional induction. In agreement, we detected lower CG methylation at the *bHLH39* promoter and lower *bHLH39* expression in *MET1*-RNA interference lines compared with wild-type seedlings. Virus-induced gene silencing of *bHLH39* and luciferase reporter assays revealed that *bHLH39* is positively involved in the modulation of Fe homeostasis. Altogether, we propose that dynamic epigenetic DNA methylation in the CG context at the *bHLH39* promoter is involved in its transcriptional regulation, thus contributing to the Fe deficiency response of tomato.

## Introduction

Iron (Fe) plays essential roles in plant survival and development. Although soils are rich in Fe, low Fe availability is common in neutral and alkaline soil conditions and results in Fe deficiency [1]. To adapt and respond to low Fe conditions, plants have evolved two intricate strategies to ameliorate Fe acquisition and uptake from the soil [2]. Briefly, in dicotyledonous non-gramineous Strategy I plants, the plasma membrane H^+^-ATPase AHA2 (ARABIDOPSIS H^+^-ATPASE 2) pumps protons into the rhizosphere to facilitate the dissolution of soluble ferric iron (Fe^3+^) by lowering the pH. Fe^3+^ is then reduced to Fe^2+^ by FRO2 (FERRIC CHELATE REDUCTASE 2) and subsequently taken up into the cytoplasm by IRT1 (IRON-REGULATED TRANSPORTER 1) [3, 4]. However, Strategy II plants solubilize Fe^3+^ by secreting phytosiderophores (PSs), after which the Fe^3+^–PS complex is transported by YELLOW STRIPE (YS) or YS-like (YSL) transporters into the root [5].

Plants maintain cellular Fe homeostasis via transcriptional regulation, whereby transcription factors (TFs) from the basic helix–loop–helix (bHLH) family assemble into a sophisticated regulatory network [6]. In the Strategy I plant species *Arabidopsis thaliana*, these bHLH TFs are divided into two core signaling pathways induced by Fe deficiency: FIT-dependent and PYE-dependent. FIT, namely, FER-LIKE IRON DEFICIENCY-INDUCED TRANSCRIPTION FACTOR, forms heterodimers with the subgroup Ib bHLH TFs bHLH38, bHLH39, bHLH100, and bHLH101, which activate the transcription of genes associated with Fe acquisition, such as *FRO2*, *AHA2*, and *IRT1* [7–9]. However, PYE (POPEYE, also named bHLH047) interacts with other IVc bHLH TFs, such as bHLH104, bHLH115, and bHLH105 [also named IAA-LEUCINE RESISTANT3 (ILR3)], to repress transcription of genes related to Fe mobilization, such as *FRO3* and *NAS4* (*NICOTIANMINE SYNTHASE 4*) [10–12]. The transcriptional network appears to be even more complex, as bHLH121 can also interact with the bHLH IVc proteins bHLH34, bHLH104, and bHLH115 to modulate the transcript abundance of bHLH Ib TF genes [13–15]. Moreover, BRUTUS, an Fe-binding E3 ubiquitin ligase, is involved in the negative regulation of Fe deficiency responses via directly interacting with ILR3, bHLH104, and bHLH115. Additionally, BRUTUS can fine-tune Fe uptake by degrading FIT through the 26S proteasome pathway [10, 16].

Accumulating evidence reveals that epigenetic factors such as DNA methylation, histone modifications, and siRNAs (small interfering RNAs) are vital for regulating gene expression and counteract stress imposed by mineral nutritional deficiency [17–21]. Under high nitrogen conditions, *Arabidopsis* HNI9 (HIGH NITROGEN INSENSITIVE 9) mediates the accumulation of H3K27me3 (trimethylation of lysine 27 in histone H3) to repress *NRT2.1* (*NITRATE TRANSPORTER 2.1*) expression and decrease nitrogen uptake [22]. Under low nitrogen conditions, the histone methyltransferase SDG8 (SET DOMAIN GROUP 8) modulates H3K36me3 abundance in response to light and/or carbon signals [23]. Moreover, in rice (*Oryza sativa*), overexpression of *OsNGR5* (rice *NITROGEN*-*MEDIATED TILLER GROWTH RESPONSE 5*) increased tiller number and improved nitrogen use efficiency via H3K27me3 modification under different nitrogen conditions [24]. In maize (*Zea mays*) root, CG methylation decreased across the entire genome under low nitrogen and phosphate conditions [25]. Phosphate deficiency can dramatically reshape DNA methylation in other plants, like *Arabidopsis* and rice [19, 20]. Additionally, histone modifications mediated by the histone deacetylase HDA19, AL6 (ALFIN-LIKE 6)-mediated H3K4me3, and HDC1 (HISTONE DEACETYLATION COMPLEX 1)-mediated H3ac (acetylation of histone H3) have been identified to participate in downstream signaling under phosphate deficiency [21, 26, 27]. Notably, a few epigenetic mechanisms involved in Fe homeostasis have been reported. For instance, SKB1 [SHK1 BINDING PROTEIN 1, also named PRMT5 (PROTEIN ARGININE METHYLTRANSFERASE 5)] negatively regulates Fe homeostasis via histone H4R3 symmetric demethylation (H4R3me2) at *bHLH38*, *bHLH39*, *bHLH100*, and *bHLH101* chromatin [28]. The histone acetyltransferase GCN5 (GENERAL CONTROL NONDEREPRESSIBLE 5) was also reported to regulate FRD3 (FERRIC REDUCTASE DEFECTIVE 3)-mediated iron homeostasis by directly binding to the *FRD3* promoter and modulating acetylation levels at H3K9 and H3K14 [29]. In addition, Polycomb Repressor Complex 2 (PRC2)-mediated H3K27me3 directly targets *FIT* and the FIT target genes *IRT1* and *FRO2* to modulate their expression under Fe deficiency [30]. Moreover, NRF2 (NON-RESPONSE TO Fe-DEFICIENCY 2), also named ELF8 (EARLY FLOWERING 8), was identified as being essential for *GRF11* (GENERAL REGULATORY FACTOR 11) transcriptional activation via H3K4me3, thereby regulating Fe homeostasis [31].

A function for DNA methylation in Fe homeostasis has seldom been reported. Methylation-sensitive amplified polymorphism analysis demonstrated that Fe deficiency can induce significant changes in the DNA methylome in barley (*Hordeum vulgare*) [32]. Comparative methylome and transcriptome analyses indicated that Fe deficiency induced the hypermethylation of two crucial bHLH TF genes, *OsIRO2* (rice *IRON*-*RELATED bHLH TRANSCRIPTION FACTOR 2*) and *OsbHLH156*, which affect most Fe deficiency responses in rice [33]. Importantly, these examples concentrated on Strategy II plants. However, in Strategy I plants the relationship between DNA methylome and transcriptional reprogramming under Fe deficiency remains to be investigated. In this study, by analyzing the global changes of DNA methylation patterns and the transcriptome of tomato (*Solanum lycopersicum*), a typical Strategy I plant, in response to both short-term (12 hours) and long-term (72 hours) Fe deficiency, we dissected the dynamics of the DNA methylome in response to Fe deficiency and demonstrated that CG hypermethylation of the *bHLH39* promoter regulates its transcription, which contributes to Fe deficiency responses of tomato roots.

## Results

### Treatment with a methyltransferase inhibitor represses Fe deficiency-induced responses

**Figure 1:**
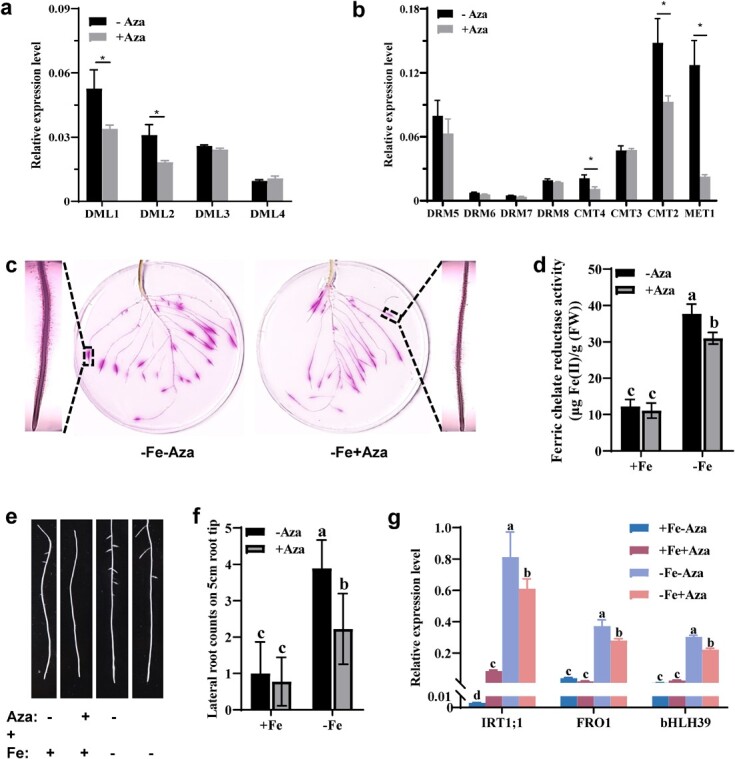
Effects of DNA methylation inhibitor in tomato roots under Fe-deficient conditions. **a**, **b** Expression of genes involved in DNA methylation/demethylation after Aza treatment under Fe-sufficient conditions. Aza (5-aza-2-deoxycytidine) was used here as a DNA methylation inhibitor with a concentration of 5 μM. **c**, **d** Root FCR localization (**c**) and root relative FCR activity (**d**) under Fe deficiency with or without 5 μM Aza treatment for 3 days. **e**, **f** Phenotype of lateral root (**e**) and statistical analysis of lateral root numbers (**f**) under Fe sufficiency or deficiency treated with 5 μM Aza. **g** Transcript abundance of three core genes induced by Fe deficiency (*IRT1;1*, *FRO1* and *bHLH39*) in response to Aza treatment under Fe-sufficient or Fe-deficient conditions. Data are mean ± standard deviation (*n* = 5 for FCR activity, *n* = 9 for lateral root number, *n* = 3 for gene expression) followed by different letters or asterisks indicating a statistical difference at *P* ≤ .05 level.

To test whether changes in DNA methylation affect the Fe deficiency responses of a Strategy I plant like tomato [cultivar ‘Ailsa Craig (AC)], we used the DNA methylation inhibitor 5-azacytidine (Aza) to investigate its influence on the expression of Fe deficiency-induced responses. To minimize the adverse effects of Aza on tomato seedlings, we determined the optimal concentration for this chemical. We observed that exogenous application of Aza at a concentration as low as 5 μM resulted in a slight inhibition of primary root elongation and lower shoot biomass; these adverse effects became more pronounced with higher Aza concentrations (Supplementary Data Fig. S1). Moreover, the expressions of the methylase-related genes *DML1* (*DEMETER*-*LIKE 1*), *DML2*, *CMT2* (*CHROMOMETHYLASE 2*), *CMT4*, and *MET1* (*METHYLTRANSFERASE 1*) were strongly repressed by 5 μM Aza treatment even under Fe sufficiency (Fig. 1a and b). Therefore, we used 5 μM Aza here.

Given that the induction of root ferric chelate reductase (FCR) activity and development of root morphology reflect Fe deficiency responses in tomato [34, 35], we evaluated the effects of 5 μM Aza on these physiological and morphological responses under Fe deficiency conditions. The induction of root FCR activity was greatly attenuated upon Aza treatment, as was the Fe deficiency-induced development of root hairs and lateral roots (Fig. 1c−f). Consistently, the transcript levels of three core genes, *IRT1;1*, *FRO1*, and *bHLH39*, were also slightly lower under combined Fe deficiency and exogenous Aza application than in seedlings only exposed to Fe deficiency (Fig. 1g). These results indicate that DNA methylation may be implicated in regulating Fe deficiency-induced responses in tomato roots.

### Global DNA methylation profiling of roots exposed to Fe deficiency

**Figure 2:**
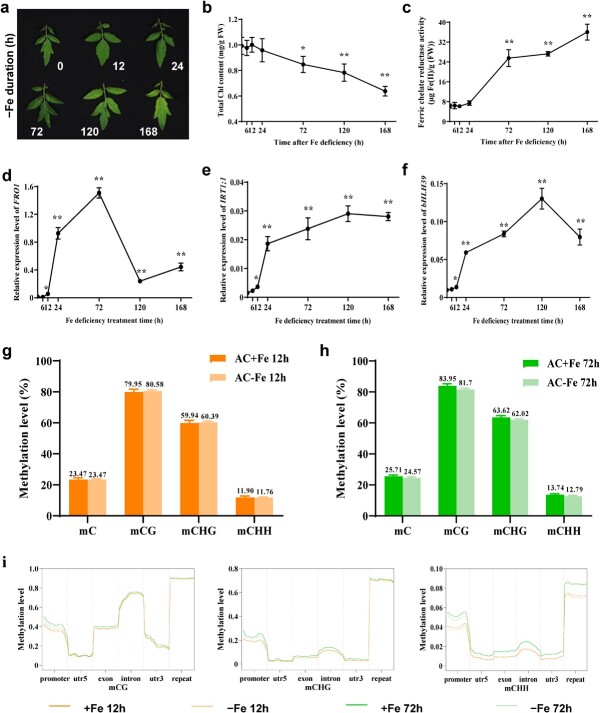
Effects of Fe deficiency on global DNA methylation in tomato root. **a**–**c** Phenotype of chlorotic leaves (**a**), total Chl content (**b**), and relative root FCR activity (**c**) in AC under Fe deficiency. **d**–**f** Time-course expression of *FRO1* (**d**), *IRT1;1* (**e**), and *bHLH39* (**f**) in tomato roots under Fe deficiency. Seedlings of WT tomato (AC) were supplied with 0 μM Fe-EDTA (−Fe) in culture solution for 0, 12, 24, 72, 120 and 168 hours. Data are mean ± standard deviation (*n* = 10 for total Chl content, *n* = 3 for gene expression). Asterisks indicate significant differences compared with the 0-hour control by Turkey’s test (^*^P ≤ .05, ^**^P ≤ .01). **g**, **h** Bulk DNA methylation in CG, CHG, and CHH context in AC under Fe-sufficient and Fe-deficient conditions for 12 hours (**g**) and 72 hours (**h**). **i** Methylation level of mCG, mCHG, and mCHH occurred at promoter, 5′-UTR, exon, intron, 3′-UTR, and repeat regions. Lines with different colors represent AC under Fe-sufficient and Fe-deficient conditions for 12 or 72 hours.

To examine the global effects of Fe deficiency on DNA methylation in tomato roots, we carried out a time-course experiment using AC seedlings. Fe deficiency often leads to interveinal chlorosis [4], but we only started to observe this phenotype after at least 72 hours of growth in Fe-deficient conditions (Fig. 2a; Supplementary Data Fig. S2). Indeed, total chlorophyll (Chl) contents were significantly reduced after a minimum of 72 hours of Fe deficiency (Fig. 2b). Consistent with this observation, root FCR activity started to increase between 24 and 72 hours of Fe deficiency treatment, after which FCR activity remained high until 168 hours (Fig. 2c). We also analyzed the transcript levels of the three marker genes *FRO1*, *IRT1;1*, and *bHLH39*, which showed a slight induction after 12 hours of Fe deficiency, but rose to much higher levels thereafter (Fig. 2d–f). Interestingly, *FRO1* expression, whose encoding enzyme contributes to root FCR activity, peaked at 72 hours before declining (Fig. 2d), whereas *IRT1;1* and *bHLH39* transcript levels reached their highest levels at 120 hours and then decreased (Fig. 2e and f). Collectively, these results suggest that changes in transcript levels are more rapid than physiological responses in generating visible phenotypic effects in tomato seedlings and that at least 12 hours of growth under Fe deficiency is required for tomato seedlings to initiate the transcriptional cascade that activates Fe-responsive genes.

Based on the analysis above, we selected 12 and 72 hours of Fe deficiency for short-term and long-term treatment, respectively, of tomato roots for whole-genome bisulfite sequencing (WGBS). We obtained 30 Gb clean bases for each sample with a conversion rate of 99.6% and then mapped the clean reads to the tomato reference genome (Supplementary Data Table S2). Altogether, we identified 733 116 220 mapped total cytosine sites across the genome, with an average sequencing depth of 23.2× (21.22× to 25.35×). For each sample, >85% of the sequenced sites reached a coverage level of at least 10× (Supplementary Data Table S2). The large correlation coefficients of three biological replicates indicated the high reproducibility for each treatment (Supplementary Data Fig. S3). These results demonstrate the high quality of our WGBS data. Among aligned methylated cytosines (^m^C) in all three sequence contexts (^m^CG, ^m^CHG, and ^m^CHH), Fe deficiency caused no obvious alterations after short-term treatment, although we noticed a slight decrease in methylation after long-term treatment (Fig. 2g and h). However, when assessing DNA methylation patterns in different genomic regions, we observed that methylation levels were significantly higher in all samples collected after 72 hours compared with those collected after 12 hours, irrespective of Fe status, but the demethylation induced by Fe deficiency was stronger at 72 hours than at 12 hours, especially at promoter regions (Fig. 2i; Supplementary Data Fig. S4).

**Figure 3:**
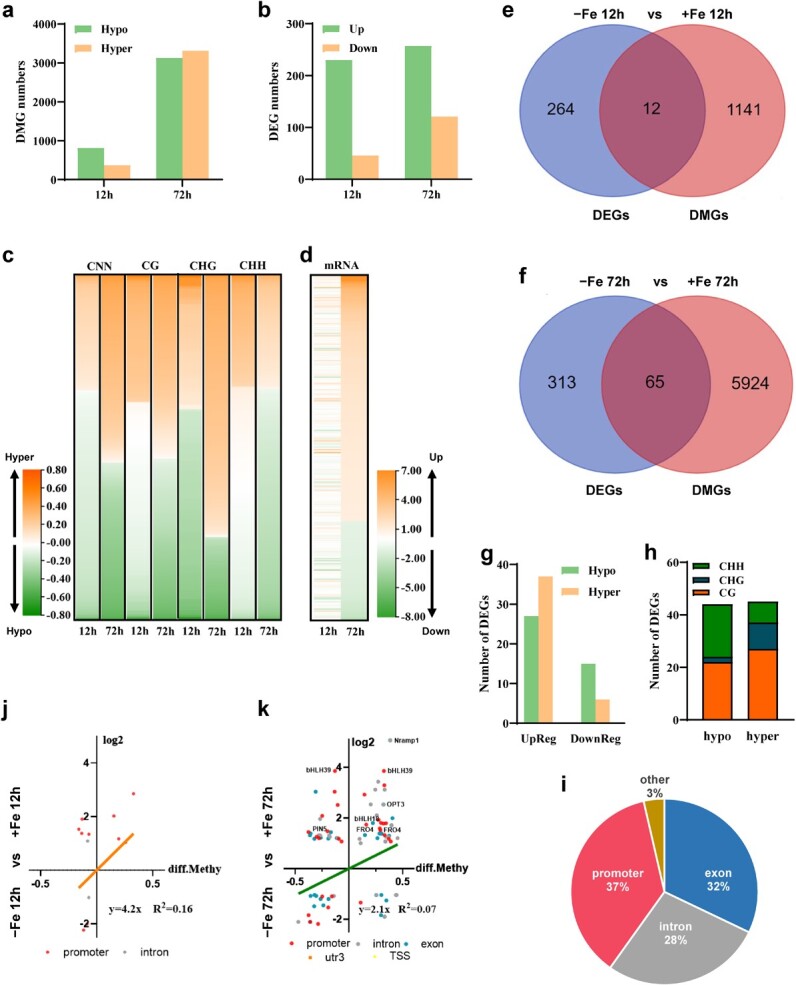
Combinational analyses of DMGs and DEGs under Fe deficiency. **a**, **b** Number of hyper-/hypo-DMGs (**a**) and up/downregulated DEGs (**b**) in tomato roots subjected to Fe-deficient conditions. Wild-type tomato seedlings were treated under Fe-sufficient and Fe-deficient conditions for 12 or 72 hours. **c**, **d** Heat maps of Fe-deficiency-induced DMRs in three cytosine contexts (**c**) and DEGs in response to Fe deficiency (**d**). Scale bars in (**c**) show the different methylation levels of DMRs and the scale bars in (**d**) show fold changes in DEGs which have different clustering partners. **e**, **f** Venn diagrams displaying the association between DMGs and DEGs under Fe deficiency for 12 hours (**e**) or 72 hours (**f**). **g** Upregulated and downregulated genes associated hyper-/hypo-methylated regions under Fe-deficient conditions for both treatment times. **h**, **i** DMGs associated with DEGs mapped to different context (**h**) and gene features (**i**). **j**, **k** Scatter plot between methylation and transcription under Fe-sufficient and Fe-deficient conditions for 12 hours (**j**) or 72 hours (**k**).

To evaluate the effects of dynamic changes in DNA methylome on gene expression, we screened differentially methylated regions (DMRs) and DMR-associated genes (DMGs), and identified 2510 and 10 657 DMRs under short- and long-term Fe deficiency conditions, respectively (Supplementary Data Tables S3 and S4). Notably, the most significant changes in DMRs occurred at CG sites (3008 hyper-DMRs and 2536 hypo-DMRs) (Supplementary Data Fig. S5a and b). Among these DMRs, we detected 1153 DMGs (811 DMGs associated with hypo-DMRs and 367 DMGs associated with hyper-DMRs) in the short-term treatment samples, and 5989 DMGs (3128 DMGs associated with hypo-DMRs and 3312 DMGs associated with hyper-DMRs) in the long-term treatment samples, compared with Fe-sufficient conditions (Fig. 3a; Supplementary Data Tables S5 and S6). Most DMGs showed that differential methylation dominated at CHH sites during short-term treatment but at CG sites during long-term treatment (Supplementary Data Fig. S5c).

### Linkage between DNA methylome and RNA transcriptome

To connect DNA methylation changes with transcriptional regulation, we carried out RNA sequencing (RNA-seq) analysis under the same treatment conditions. We identified 276 and 378 differentially expressed genes (DEGs) after short- and long-term Fe deficiency treatment of tomato roots, respectively (Fig. 3b; Supplementary Data Tables S7 and S8). Only 34 DEGs overlapped between the two treatments (Supplementary Data Fig. S6a; Supplementary Data Table S9). While most DEGs showed differential expression patterns over time, some genes, such as *bHLH101*, *bHLH100*, *bHLH39*, *IRT1;1*, and *OPT3* (*OLIGOPEPTIDE TRANSPORTER 3*), showed increased expression with longer treatment time (Supplementary Data Fig. S6b). Overall, the number of hyper-DMRs increased over time, especially in the CG and CHG contexts (Fig. 3c), which was accompanied by greater transcript levels of Fe deficiency-induced genes (Fig. 3d).

To further examine the interconnection between methylation changes and gene expression, we looked for DEGs that are induced by Fe deficiency and related to DMGs. We identified 12 and 65 DMG–DEGs in response to short- and long-term Fe deficiency, respectively (Fig. 3e and f; Supplementary Data Tables S10 and S11). Among these DMG–DEGs, 27 hypo-DMGs and 37 hyper-DMGs were upregulated, and 15 hypo-DMGs and 6 hyper-DMGs were downregulated (Fig. 3g). Moreover, the most abundant change occurred in the CG context among these DMG–DEGs (Fig. 3h). We classified these DMG–DEGs according to their different methylation regions, which revealed that methylation regions are mainly located in the promoter regions (37%), exons (32%), and introns (28%) of DMG–DEGs (Fig. 3i). However, we detected no obvious correlation between changes in DNA methylation levels of DEGs and changes in their transcript levels in either short-term (*R*^2^ = 0.16) or long-term (*R*^2^ = 0.07) treatments (Fig. 3j and k), with the exception of five genes: *bHLH39*, *bHLH18*, *Nramp1* (*NATURAL RESISTANCE-ASSOCIATED MACROPHAGE PROTEIN 1*), *OPT3*, and *FRO4*, whose expression changes displayed some relationships with DNA methylation changes under long-term treatment (Fig. 3k). Among these five genes, DNA methylation changes in *bHLH39* and *bHLH18* occurred over their promoter regions, while those in *Nramp1*, *OPT3*, and *FRO4* were mainly concentrated on the gene body (Table 1).

**Table 1:** Gene expression and DNA methylation changes in several known Fe deficiency-responsive mark genes in tomato.

Gene name	Gene ID	log_2_FC (12 hours)	log_2_FC (72 hours)	differentially methylated level (72 hours)	^m^C_context	Region	Start	End
*bHLH104*	Solyc09g089870.2	0.04	−0.19	−0.11	CHH	Promoter	69 496 369	69 496 481
				−0.23	CHG	Promoter	69 497 576	69 497 669
				−0.32	CHG	Promoter	69 496 357	69 496 455
				−0.35	CG	Promoter	69 496 323	69 496 380
				−0.36	CG	Promoter	69 497 512	69 497 686
*bHLH39*	Solyc10g079650.1	1.06	3.86	0.33	CG	Promoter	61 150 224	61 150 286
				−0.13	CHH	Promoter	61 150 891	61 150 975
*bHLH18*	Solyc02g091820.1	2.06	1.81	0.30	CG	Promoter	53 040 271	53 040 325
*Nramp1*	Solyc11g018530.1	0.22	5.07	0.38	CG	Intron	8 643 310	8 643 372
				1.38	CG	Exon	8 643 310	8 643 372
*OPT3*	Solyc11g012700.1	1.75	2.54	0.33	CG	Intron	5 471 137	5 471 326
				1.33	CG	Exon	5 471 137	5 471 326
*FER*	Solyc05g052470.2	−0.36	−0.51	−0.13	CHH	Promoter	62 684 990	62 685 056
				−0.16	CHH	Promoter	62 684 685	62 684 805
*FRO4*	Solyc00g020840.2	−2.16	1.33	0.33	CHG	Promoter	11 899 762	11 899 917
				0.15	CG	Exon	11 901 672	11 901 744

**Figure 4:**
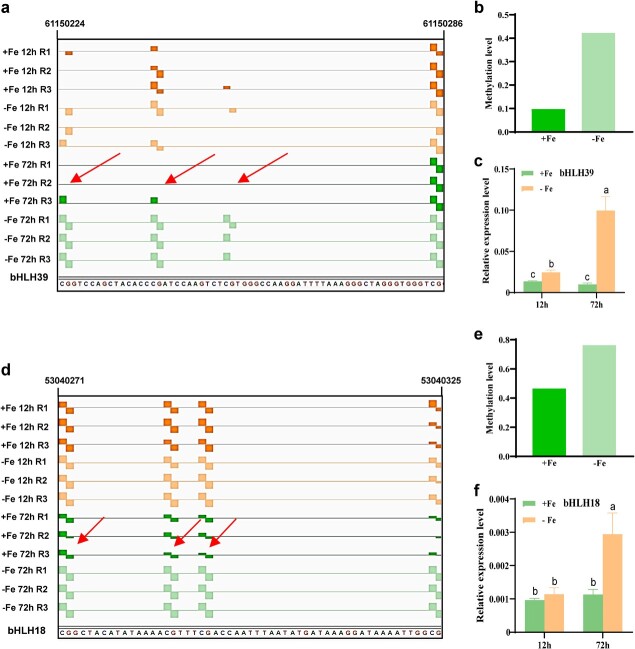
Two core Fe deficiency induced genes presented the relationship between DNA methylation and gene expression by genome browser. **a**–**c** Methylation level of *bHLH39* (**a**, **b**) and its gene expression (**c**). **d**–**f** Methylation level of *bHLH18* (**d**, **e**) and its gene expression (**f**). After growing in Fe-sufficient or Fe-deficient solution for 12 and 72 hours, AC roots were harvested for DNA and RNA extraction. The methylation level was analyzed by profiling of DNA methylation. RT–qPCR was used to detect gene expression with *ACTIN* as an internal control. Data are mean ± standard deviation (*n* = 3 biological repeats). Different letters indicate a statistical difference among different treatments by Turkey’s test at the level of *P* ≤ .05.

We further explored the linkage between methylation changes and gene expression in terms of DMRs and different methylation contexts. We observed no global correlation between differential DNA methylation and mRNA abundance, except in the CG context when located in the promoters of DMG–DEGs (Supplementary Data Fig. S7). While most methylation changes occurred in the CG context at both exons and introns of DMG–DEGs (Supplementary Data Fig. S7a, d, and g), methylation changes in the contexts of CG and CHH predominated in their promoter regions (Supplementary Data Fig. S7g and i). Spearman correlation coefficient analysis indicated that the correlation of methylation with transcription in promoter regions was significantly higher than that with exons or introns (Supplementary Data Fig. S7j–l), suggesting a potential correlation between promoter methylation changes and transcriptional regulation of related genes. In particular, although the transcript changes of few genes were significantly correlated to their methylation changes, two TF genes, *bHLH39* and *bHLH18*, which activate the transcription of Fe deficiency-responsive genes [6–8], were both hypermethylated preferentially in the CG context at their promoters (Supplementary Data Fig. S7g). We observed more hypermethylation than hypomethylation at CG sites in the *bHLH39* promoter, which was associated with the induction of *bHLH39* expression by long-term Fe deficiency treatment (Fig. 4a and b). Additionally, while *bHLH18* expression indeed increased under long-term Fe deficiency treatment, the closest hypermethylated region was very far from its transcription start site (~638 691 bp) and is thus very unlikely to regulate gene expression (Fig. 4c and d). Altogether, we showed that promoter CG hypermethylation at *bHLH39* is related to transcriptional induction by Fe deficiency.

**Figure 5:**
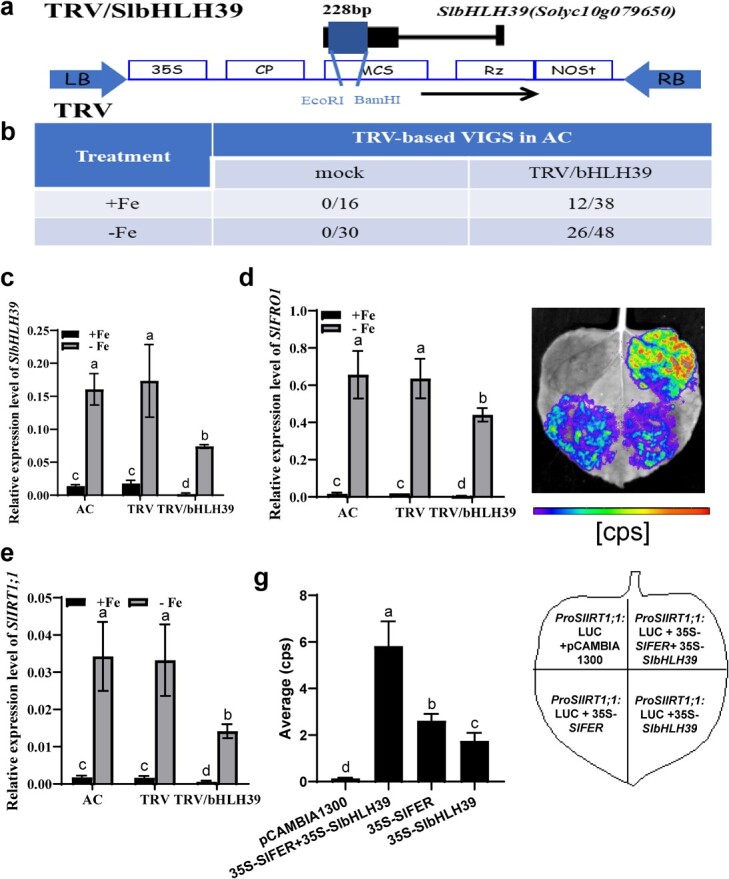
Function analysis of *bHLH39* in tomato. **a** Schematic diagram of *bHLH39* and the TRV-based VIGS construct TRV/bHLH39. **b** Summary of TRV-induced silencing efficiency in AC plants. Numerators represent the number of silenced plants, as determined by measuring the expression of *bHLH39*. Denominators represent the total number of plants per treatment. **c**–**e** Suppressed expression of endogenous *bHLH39* (**c**), *FRO1* (**d**) and *IRT1;1* (**e**) in *bHLH39*-silenced line. **f**, **g** Transient transcriptional activity assays verifying that bHLH39, like FER, regulated *IRT1;1* promoter activity. The colored scale bar indicates the luminescence intensity (**f**), and relative luminescence intensities are shown in (**g**). Mean ± standard deviation (*n* = 10), with different letters indicating significant differences among treatments at *P* ≤ .05.

To prove that bHLH39 is a key factor implicated in Fe deficiency-inducible responses, we performed tobacco rattle virus (TRV)-induced *bHLH39* silencing in AC plants. Compared with control seedlings, the expression of endogenous *bHLH39* was downregulated by ~60–90% (Fig. 5a−c). Furthermore, the mRNA abundances of two major Fe uptake genes, i.e. *FRO1* and *IRT1;1*, were also decreased in *bHLH39*-silienced lines (Fig. 5d and e), implying the importance of bHLH39 in the modulation of Fe homeostasis. Moreover, we carried out a transient gene-expression assay by co-expressing FER, bHLH39 and the targeted promoter of *IRT1;1* in *Nicotiana benthamiana* leaves. As shown in Fig. 5f and g, either bHLH39 or FER activated *IRT1;1* promoter activity, and this activation could be further enhanced by the presence of both bHLH39 and FER. These observations suggest the participation of bHLH39 in regulating Fe homeostasis in tomato.

### DNA demethylation alters Fe homeostasis

**Figure 6:**
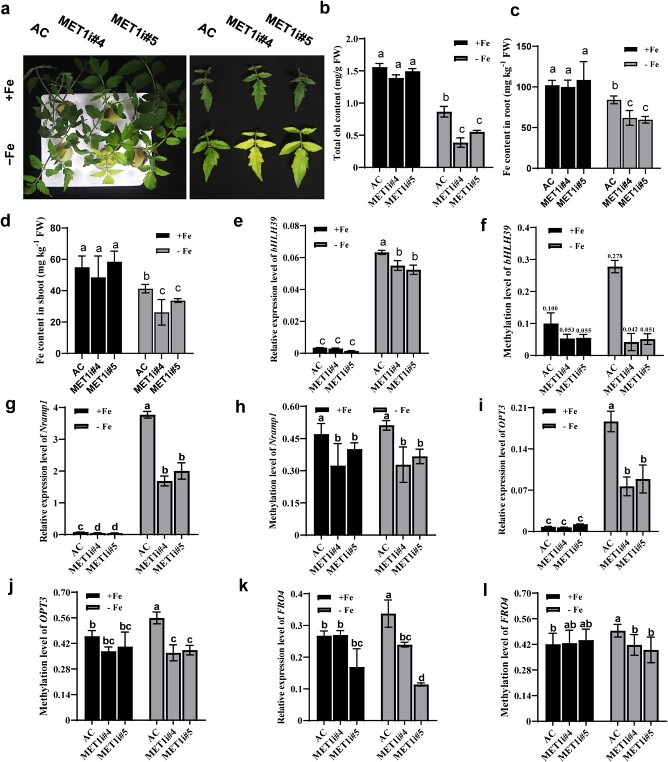
Silencing of *MET1* alters Fe deficiency-induced responses in tomato. **a**, **b** Phenotype of tomato leaves (**a**) and total Chl content (**b**) in both *MET1*-RNAi lines subjected to Fe-deficient conditions for 9 days. **c**, **d** Fe content in roots (**c**) and shoots (**d**) of AC and both *MET1*-RNAi lines in response to Fe deficiency. After a 9-day treatment, both roots and shoots were harvested, dried, and digested for Fe analysis by ICP–OES. **e**, **g**, **i**, **k** Expression analysis of *bHLH39*, *Nramp1*, *OPT3*, and *FRO4* in response to Fe deficiency, respectively. **f**, **h**, **j**, **l** Targeted bisulfite sequencing shows the DNA methylation level of *bHLH39*, *Nramp1*, *OPT3*, and *FRO4* within CG-type DMRs of these in AC and both *MET1*-RNAi lines. Roots of AC and both *MET1*-RNAi lines (*MET1i#4* and *MET1i#5*) grown in Fe-sufficient or Fe-deficient solutions for 9 days were harvested for gene expression and targeted bisulfite sequencing. Mean ± standard deviation (*n* = 12 for leaf Chl content, *n* = 7 for Fe content, *n* = 3 for gene expression, *n* = 3 for targeted bisulfite sequencing) with different letters indicating significant differences among treatments at the *P* ≤ .05 level.

To confirm that CG hypermethylation of the *bHLH39* promoter is associated with its higher expression under Fe deficiency, we tested the interconnection between maintenance of CG methylation and Fe deficiency-induced responses in two independent *MET1*-RNAi lines, in which *MET1* expression was significantly lower [36]. MET1 maintains CG methylation once established by DRM2 (DOMAINS REARRANGED METHYLTRANSFERASE 2) through the RNA-directed DNA methylation pathway [37]. Compared with AC seedlings, *MET1*-RNAi lines displayed a more severe chlorosis phenotype (Fig. 6a), which we confirmed by its lower Chl contents (Fig. 6b) and lower Fe contents under Fe deficiency (Fig. 6c and d), demonstrating that global alterations in CG methylation levels affect Fe homeostasis in tomato.

Next, we examined the promoter CG methylation levels and the expression of *bHLH39* in the *MET1*-RNAi lines. The expression of *bHLH39* was lower in *MET1*-RNAi lines under the same conditions (Fig. 6e). Concurrently, the region of the *bHLH39* promoter that was hypermethylated in AC was demethylated in the two *MET1*-RNAi lines under Fe deficiency (Fig. 6f). Therefore, a decrease in CG methylation was associated with lower *bHLH39* transcript levels of *bHLH39* in *MET1*-RNAi lines, which further confirmed the positive interconnection between promoter CG methylation and gene transcription.

In addition to *bHLH39*, there were changes in gene body CG methylation for *Nramp1*, *OPT3*, and *FRO4* in the methylation–transcription correlation analysis (Fig. 3k; Table 1). Therefore, we characterized the DNA methylation and gene expression levels of *Nramp1*, *OPT3*, and *FRO4* in *MET1*-RNAi lines*.* Compared with the wild type (WT), DNA methylation levels at *Nramp1*, *OPT3*, and *FRO4* were lower in *MET1*-RNAi lines under Fe-deficient conditions. Moreover, the higher expression of *Nramp1*, *OPT3*, and *FRO4* upon Fe deficiency seen in AC tomato seedlings was repressed in *MET1*-RNAi lines (Fig. 6g−l). These results reveal that CG methylation, especially at the *bHLH39* promoter, is associated with transcriptional induction to regulate Fe deficiency responses.

## Discussion

The molecular and genetic mechanisms regulating Fe homeostasis have been extensively examined by determining plant responses to Fe deficiency [5]. However, limited information is available regarding the potential for epigenetic regulation of gene expression under Fe deficiency. Three classic epigenetic mechanisms, i.e. histone modifications, DNA methylation, and non-coding RNAs (ncRNAs), are associated with gene expression regulation [17, 38]. Previous studies have linked histone post-translational modifications to Fe homeostasis [28–31]. Recently, *Colorless Non*-*ripening* (*Cnr*), a tomato mutant with a hypermethylated epigenome, showed enhanced responses to Fe deficiency [39]. In this study we further explored the role of dynamic DNA methylation in Fe deficiency responses and implicated differential CG methylation in Fe deficiency-induced transcriptional changes.

We detected dynamic changes in DNA methylation during Fe deficiency in tomato. The most remarkable observation was that changes in DNA methylation were closely related to Fe deficiency-induced responses, which is supported by the following evidence. First, changes in the DNA methylome were highly dependent on the duration of Fe deficiency treatment. Although Fe deficiency hardly affected DNA methylation levels globally, the number of DMRs increased dramatically with the extension of Fe-deficient treatment, but the distribution and pattern of DNA methylation also changed significantly (Fig. 2). For instance, 2510 DMRs occurred predominately in the CHH context after short-term Fe deficiency treatment, whereas 10 657 DMRs mainly affected the CG context upon long-term Fe deficiency treatment (Supplementary Data Tables S3 and S4). A study on the effect of nitrogen deficiency in rice also found that DNA methylation patterns changed in response to limited nitrogen status and that enhanced tolerance to nitrogen deficiency extended to the progeny of nitrogen-limited plants [40]. Recent studies have shown an apparent increase in global DNA methylation and the detection of abundant DMRs in *Arabidopsis* roots upon cadmium exposure and in tomato seedlings upon inorganic phosphate (Pi) starvation, respectively [41, 42]. Second, Fe deficiency responses were associated with changes in the DNA methylome, as evidenced by the findings that Aza, a DNA methyltransferase inhibitor, altered Fe deficiency responses (Fig. 1; Supplementary Data Fig. S1). More convincing evidence came from the *MET1*-RNAi lines, which were characterized by altered Fe deficiency responses compared with WT plants (Fig. 6). Similarly, *ddc*, a *drm1 drm2 cmt3* triple mutant with massive loss of non-CG methylation, exhibited increased sensitivity to Pi deficiency in *Arabidopsis* [19]. The root methylome was also demonstrated to respond specifically to zinc (Zn) deficiency to better utilize Zn under limited Zn status [18].

However, there was a poor correlation between changes in DNA methylation levels and changes in gene transcript levels in tomato roots exposed to either short- or long-term Fe deficiency (Fig. 3j and k). At present, reports on the interconnection between dynamic DNA methylation and gene transcription remain controversial. Some studies have suggested that DNA methylation is concerned with gene transcription changes in a dose-dependent or dynamic manner [43, 44], whilst others found no association. For example, Fan *et al*. [41] failed to detect a correlation between changes in DMRs and gene expression induced by cadmium toxicity. Limited contribution of DNA methylation to gene transcription has also been reported [45, 46].

Here we established a positive relationship between methylation changes and the transcript abundance of some core Fe deficiency-induced genes, although we detected no global correlation between the methylome and transcriptome. Upregulated Fe deficiency-responsive marker genes (*bHLH39*, *bHLH18*, *Nramp1*, *OPT3*, and *FRO4*) showed CG hypermethylation under long-term Fe deficiency (Fig. 3k; Table 1). Moreover, we also demonstrated that *bHLH39* was indeed involved in the regulation of Fe homeostasis in tomato roots (Fig. 5). Additionally, DNA demethylation decreased the expression levels of some Fe homeostasis-related genes, like *bHLH39*, *Nramp1*, *OPT3*, and *FRO4*, which we further verified in *MET1*-RNAi lines exposed to Fe deficiency (Fig. 6g–l). Considering that bHLH39 is essential in regulating Fe deficiency responses and Fe uptake [7, 8], we speculate that the lower transcript levels of *bHLH39* mainly contribute to the altered Fe deficiency responses seen in the *MET1*-RNAi lines (Fig. 6e and f). Similarly, several previous studies showed that DNA methylation affected the expression of marker genes involved in nutrient deficiency responses, such as the Pi starvation-responsive gene *SPX2* (*SPX DOMAIN GENE 2*), the Zn deficiency-responsive members from the *ZIP* (*ZRT1*, *IRT1-RELATED PROTEIN*) gene family, and Fe deficiency-inducible genes like *OsIRO2* and *OsbHLH156* [33, 18–20]. All these results indicate that DNA methylation plays a vital role in response to nutrient deficiency stress.

DNA hypermethylation at promoters is generally associated with transcriptional repression, but it can promote expression in some cases [47]. Recently, CHH methylation at promoters was shown to be associated with the upregulated expression of genes involved in fatty acid and jasmonate biosynthesis [48]. Here we demonstrated that hypermethylation of a subset of DMG–DEGs in promoters, especially in the CG context, exhibited a positive correlation with gene expression, i.e. CG hypermethylation correlated with upregulated gene expression, as with *bHLH18* and *bHLH39* (Supplementary Data Fig. S7). Moreover, CG hypomethylation resulting from the knockdown of *MET1* transcript levels in *MET1*-RNAi lines showed repressed expression of *bHLH39* (Fig. 6e and f), which strengthened the positive link between promoter DNA methylation and gene expression [47]. Additionally, CG methylation over the gene body was positively associated with gene expression. For example, we detected Fe deficiency-induced CG hypermethylation in the gene body for *Nramp1*, *OPT3*, and *FRO4* (Fig. 6g, j and l), which is consistent with a report that methylation of exon/intron occurs preferentially in the CG context [49]. Methylation within gene body regions has been associated with transcriptional upregulation and is thought to be indispensable in protecting genes from cryptic promoter-induced transcriptional abnormalities [47, 49]. MET1 is well known for establishing methylation in the predominantly occurring CG context. Systematic analysis of global expression profiles in different *Arabidopsis* accessions knocked out for *MET1* by gene editing revealed diverse relationships between CG methylation and gene expression [50]. Our results revealed the correlation between methylation and transcription in several Fe deficiency-responsive marker genes; however, how the global CG methylation changes affect gene expression under Fe deficiency requires to be further investigated in the future.

In summary, we revealed the dynamics of both DNA methylation and the transcriptome, which is important for Fe deficiency-induced responses in tomato roots. Additionally, DNA demethylation imposed either via a chemical inhibitor of DNA methyltransferase activity or *MET1*-RNAi showed that dynamic alterations of DNA methylation are necessary for the proper induction of Fe deficiency responses. By combining methylome analysis and a genetic approach, we propose that DNA methylation in the CG context is positively related to gene expression and mediates the transcription of specific genes, such as *bHLH39*, which contributes to Fe deficiency responses in tomato roots.

## Materials and methods

### Plant materials and growth conditions

*Solanum lycopersicum* WT cultivar ‘Ailsa Craig’ (AC) and the *MET1*-RNAi transgenic lines [36] were used here. Surface-sterilized tomato seeds were sown on one-fifth-strength Hoagland solution (pH 5.5) solidified with 1% (w/v) agar. When the primary roots were ~3–4 cm in length, uniform seedlings were transplanted to one-fifth Hoagland nutrient hydroponic solution for acclimation. Tomato seedlings at the second true leaf stage were grown in one-fifth-strength Fe-free Hoagland nutrient solution supplemented with either 20 μM (Fe sufficiency, +Fe) or 0 μM Fe-EDTA (Fe deficiency, −Fe) for 0, 6, 12, 24, 48, 72, 120, or 168 hours. For exogenous treatment with Aza, a DNA methylation inhibitor, 5, 10, or 20 μM Aza was added to the +Fe or −Fe growth medium for 3 days. Roots were harvested and quickly frozen in liquid nitrogen for further analysis.

### Leaf morphological analysis and Fe determination

Since no obvious changes in chlorosis were visible after −Fe treatment for 6 hours, young chlorotic leaves under −Fe conditions were photographed at 0, 12, 24, 48, 72, 120, and 168 hours using a Nikon digital camera. To estimate the Chl content, leaves were immersed in 80% (v/v) acetone in darkness until completely bleached and then the contents of Chl *a* and Chl *b* were measured with a spectrophotometer at 663 and 646 nm, respectively. Separated roots and shoots were weighed. After drying, the samples were digested in concentrated HNO_3_ for 5 hours at 160°C, and their mineral contents were determined by ICP–MS (inductively coupled plasma–mass spectrometry; Agilent 7500ce, USA) as previously described [35, 39].

### Measurement of root morphology and root ferric chelate reductase activity

After a 3-day Fe-deficient treatment combined with exogenous Aza application, root morphology was recorded under a microscope (Nikon Eclipse E600), and root tips 5 cm in length were used to score the number of lateral roots and for subsequent FCR assays. According to our previous study [39], tomato roots were incubated in an assay solution (pH 5.5) comprising 0.1 mM MES, 0.1 mM BPDS (bathophenanthroline disulfonate), 0.5 mM CaSO_4_, and 100 mM Fe-EDTA. After a 1-hour incubation in darkness at 25°C, the absorbance was spectrophotometrically measured at 535 nm. The data are shown as mean FCR activity relative to that in WT roots. To visualize FCR localization, roots were transferred onto plates containing the above FCR assay solution and 0.75% (w/v) agar, and photographed after 20 minutes of incubation.

### Total RNA extraction and reverse transcription quantitative real-time qPCR analysis

Total RNA from root tissues was extracted, and first-strand cDNAs were synthesized with a FastQuant RT Kit with gDNA Eraser from Tiangen, China. The subsequent reverse transcription quantitative real-time PCR (RT–qPCR) with gene-specific primers (Supplementary Data Table S1) was performed using SYBR Green chemistry (Toyobo) on a Roche LightCycler 480 machine. The relative expression level of specific genes was normalized to that of *ACTIN* as described previously [39].

### Transcriptome deep sequencing

Roots of AC seedlings were treated in +Fe or −Fe medium for 12 or 72 hours, and total RNA was extracted for the construction of TrueSeq sequencing libraries, which were then sequenced in PE150 mode (paired-end 150-bp reads) on the Illumina HiSeq platform. Three biological replicates were employed for each treatment. Raw RNA-seq data of high quality were aligned to version SL2.50 of the tomato reference genome (https://solgenomics.net). Values of RPKM (reads per kilobase per million mapped reads) were used to indicate mRNA abundance of genes, and DEGs and the corresponding analysis of Gene Ontology (GO) biological processes were performed according to our previous reports [35, 39].

### Genomic DNA isolation and targeted bisulfite sequencing

Genomic DNA was isolated from tomato roots with the DNeasy Plant Mini Kit (Qiagen). After checking DNA integrity, the genomic DNA was subjected to bisulfite conversion using an EZ DNA Methylation-Gold™ kit (Zymo Research). The following bisulfite sequencing PCR was carried out with gene-specific primers (Supplementary Data Table S1).

### Cytosine methyl-sequencing library generation and bisulfite-sequencing data analysis

Roots of AC seedlings grown in +Fe or −Fe solution for 12 or 72 hours were harvested for genomic DNA extraction. After bisulfite conversion as described above, sequencing libraries were constructed (Novogene Corporation, Beijing, China), and then bisulfite sequencing (BS-seq) was performed on the Illumina PE150 platform. For BS-seq data processing, quality control (QC) was performed by FastQC (fastqc_v0.11.5), after which the filtered reads were aligned to the tomato reference genome by Bismark software (version 0.16.3; https://www.bioinformatics.babraham.ac.uk/projects/bismark/). Cytosines with a false discovery rate (FDR)-corrected *P*-value <.05 were defined as methylated sites via a binomial test for ^m^C (methylated cytosine counts), non-^m^C (non-methylated cytosine counts), and *r* (non-conversion rate). Thereafter, the genome sequence was divided into bins of ~10 kb to calculate the sum of reads containing ^m^C and/or non-^m^C per bin, with the methylation level (ML) based on the fraction of ^m^C being defined as ML = ^m^C/(^m^C + non-^m^C). DSS software was used to identify DMRs, which were then converted into bigWig format for visualization.

### Virus-induced silencing of *bHLH39* in tomato

The TRV-based viral system was used for *bHLH39* gene silencing in AC plants as previously described [35]. An amplified 228-bp fragment of bHLH39 was ligated into the TRV-based vector to generate the TRV2/bHLH39 construct and then transformed into *Agrobacterium tumefaciens* GV3101. *Agrobacterium* strains carrying either the empty TRV1 + TRV2 vector or the empty TRV1 + TRV/bHLH39 construct were injected into tomato leaves. After infiltration for 7 days, the infected tomatoes were subjected to +Fe or −Fe treatment for another 3 days, and the roots were harvested to quantify *bHLH39*, *FRO1*, and *IRT1;1* expression.

### Luciferase activity reporter assay in *N. benthamiana*

The coding sequences of *bHLH39* and *FER* were separately cloned into the vector pCAMBIA1300 driven by the CaMV 35S promoter according to our previous research [35]. To generate the reporter construct *ProIRT1;1:LUC*, the *IRT1;1* promoter of 2000 bp from ATG was ligated into the pGreenII0800-LUC vector. Subsequently, FER, bHLH39, and the targeted promoter of *IRT1;1* were co-expressed in *N. benthamiana* leaves via *Agrobacterium*-mediated infiltration. Then, luciferase (LUC) activity was monitored by Night SHADE LB 985, a plant imaging system from Berthold.

## Acknowledgements

This work was supported financially by the Natural Science Foundation of Zhejiang Province (LZ22C150001) and the China Postdoctoral Science Foundation (2019 M652064).

## Author contributions

H.Z. and G.H. performed all the experiments. J.W. and J.X. participated in the bioinformatics analyses and element measurements. Y.H., L.H., and S.Z. were involved in manuscript editing. H.Z., J.Y., and W.C. conceived the research, analyzed the data, and wrote the article. All authors read and approved the final manuscript.

## Data availability

Tomato seeds of AC and *MET1*-RNAi lines were available in our laboratory. WGBS and RNA-seq datasets have been deposited in NCBI under accession numbers PRJNA813707 (WGBS), PRJNA813389 (12-hour RNA-seq), and PRJNA681103 (72-hour RNA-seq).

## Conflict of interest

The authors declare that they have no conflicts of interest.

## Supplementary data

Supplementary data are available at *Horticulture Research* online.

## Supplementary Material

Web_Material_uhad104
