# Supplementary material for: CG hypermethylation of the bHLH39 promoter regulates its expression and Fe deficiency responses in tomato roots
Source: Hortic Res. 2023 May 12;10(7):uhad104. doi: 10.1093/hr/uhad104 (PMC10419876; doi:10.1093/hr/uhad104)
Supplement: Web_Material_uhad104 [file web_material_uhad104.zip › Supplementary Figures for HR 230504-NEW.docx]

**Figure S1. Effects of DNA methylation inhibitor 5−azacytidine (Aza) on tomato seedings growth under Fe−sufficient and Fe−deficient conditions.**

**a and b**, The primary root length (a) and the biomass in shoot (b) under Fe deficiency with exogenous Aza application. Wild−type AC seedlings were grown under Fe−sufficient (+Fe) or Fe−deficient (−Fe) conditions with 0, 5, 10 and 20 µM DNA methyltransferase inhibitor (5−aza−2−deoxycytidine, Aza) for 3 d. Data are Means ± SD (n = 15 for primary root length, n = 9 for shoot biomass) followed by different letters indicate a statistical difference at p ≤ 0.05 by Tukey’s test.

**Figure S2.** **The phenotype of tomato leaves under Fe sufficiency.**

Seedlings of wild−type tomato (AC) were supplied with 20 µM FeEDTA (+Fe) in culture solution for 0, 12, 24, 72, 120 and 168 h, respectively. Subsequently, the phenotype of newly formed compound leaves in tomato was recorded by a digital camera.

**Figure S3.** **The correlations of methylome and RNA−sequencing among three biological replicates.**

**a−c**, The correlations of methylome in CG (a), CHG (b) and CHH (c). **d**, The correlations of RNA−sequencing for 12−h treatment. The correlations of RNA−sequencing for 72−h treatment were reported by Chen et al. (2022).

**Figure S4. Effects of Fe deficiency on DNA methylation patterns at promoter regions.**

**a−c**, The methylation levels of ^m^CG (a), ^m^CHG (b), and ^m^CHH (c) occurred at promoter. Lines with different colors represent wild−type tomato (AC) under Fe−sufficient and Fe−deficient conditions for 12 h or 72 h, respectively. **d−f,** The DNA demethylation of promoter ^m^CG (d), ^m^CHG (e), and ^m^CHH (f) induced by Fe deficiency at 12 h or 72 h.

**Figure S5.** **Differential methylated regions (DMRs) and DMR−associated genes (DMGs) in response to Fe deficiency.**

**a and b**, DNA methylation regions in CG, CHG, and CHH context under Fe−sufficient and Fe−deficient conditions for 12 h (a) or 72 h (b). **c**, Venn diagrams of hypo−DMGs (left) and hyper−DMGs (right) in the CG, CHG, and CHH contexts after 12 h (upper) or 72 h (down) treatment under Fe deficiency compared to Fe sufficiency. Short red lines indicate the majority of DMGs occurred at CHH sites during short−term (12 h) treatment, whilst at CG sites during long−term (72 h) treatment, respectively.

**Figure S6. Identification of Fe deficiency-responsive genes.**

**a**, Venn diagram showing 34 Fe deficiency−responsive genes between 12 h and 72 h treatment in response to Fe deficiency. **b**, Expression profiles of 76 Fe−deficient responsive genes via heat map analysis. The scale bars of heatmaps shows with log2 fold change (FC) between 12 h and 72 h treatment under Fe−deficient conditions.

**Figure S7. Correlation between DNA methylation and gene expression.**

**a**−**c**, Combinational analysis between transcript abundance and their corresponding DNA methylation within exon regions at CG (a), CHG (b) or CHH (c) sites. **d**−**f**, Combinational analysis between transcript abundance and their corresponding DNA methylation within intron regions at CG (d), CHG (e) or CHH (f) sites. **g**−**i**, Combinational analysis between transcript abundance and their corresponding DNA methylation within promotor regions at CG (g), CHG (h) or CHH (i) sites. The red spots in (g) were presented as bHLH18 and bHLH39, respectively. **j**−**l**, The correlation analysis of DNA methylation mapped to exon (j) or intron (k) or promoter (l) and gene expression by spearman correlation coefficient.
